# Supplementary material for: Epigenetic predictors of lifestyle traits applied to the blood and brain
Source: Brain Commun. 2021 Apr 19;3(2):fcab082. doi: 10.1093/braincomms/fcab082 (PMC8134833; doi:10.1093/braincomms/fcab082)
Supplement: fcab082_Supplementary_Data [file fcab082_supplementary_data.zip › Supplementary_Figure.docx]

**
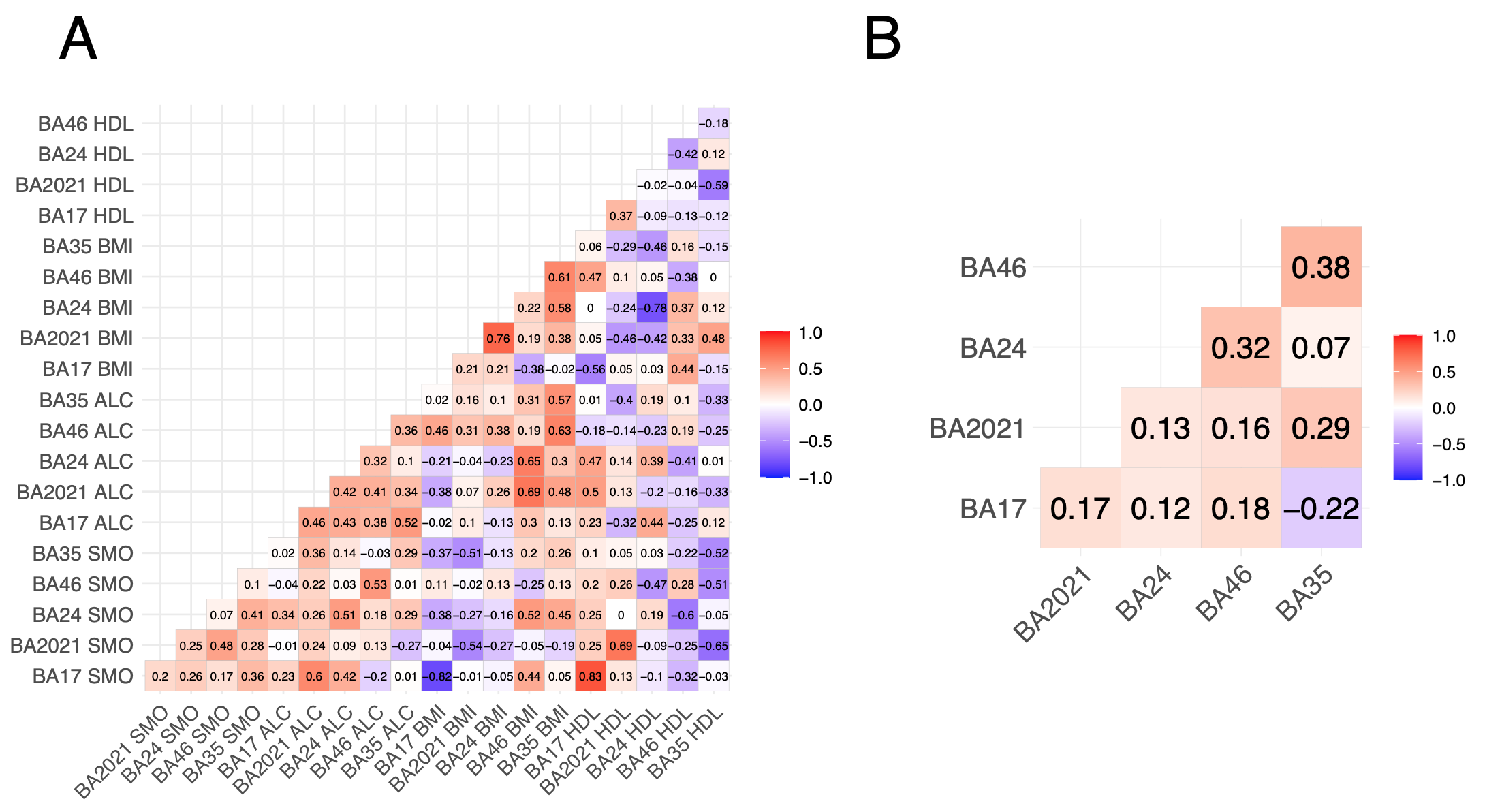
Supplementary Figure 1. Heatmaps showing correlations for (A) predictor scores and (B) DNA methylation at the CpG site cg05575921 across the five brain regions in the brain bank group.** Spearman correlation coefficients are presented in each case. There were 14 measurements available, except one individual for which hippocampal (BA35) sampling was not available. BA: Brodmann’s area. HDL: high density lipoprotein. BMI: body mass index. ALC: alcohol. SMO: smoking.
